# Five-Golden-Flowers Tea: Green Extraction and Hepatoprotective Effect against Oxidative Damage

**DOI:** 10.3390/molecules23092216

**Published:** 2018-08-31

**Authors:** Cai-Ning Zhao, Guo-Yi Tang, Qing Liu, Xiao-Yu Xu, Shi-Yu Cao, Ren-You Gan, Ke-Yi Zhang, Shuang-Li Meng, Hua-Bin Li

**Affiliations:** 1 Guangdong Provincial Key Laboratory of Food, Nutrition and Health, Guangdong Engineering Technology Research Center of Nutrition Translation, Department of Nutrition, School of Public Health, Sun Yat-Sen University, Guangzhou 510080, China; zhaocn@mail2.sysu.edu.cn (C.-N.Z.); tanggy5@mail2.sysu.edu.cn (G.-Y.T.); liuq248@mail2.sysu.edu.cn (Q.L.); xuxy53@mail2.sysu.edu.cn (X.-Y.X.); caoshy3@mail2.sysu.edu.cn (S.-Y.C.); zhangky6@mail2.sysu.edu.cn (K.-Y.Z.); mengshl9@mail2.sysu.edu.cn (S.-L.M.); 2 Department of Food Science & Technology, School of Agriculture and Biology, Shanghai Jiao Tong University, Shanghai 200240, China; renyougan@sjtu.edu.cn; 3 South China Sea Bioresource Exploitation and Utilization Collaborative Innovation Center, Sun Yat-Sen University, Guangzhou 510006, China

**Keywords:** herbal tea, antioxidant activity, polyphenol, microwave-assisted extraction, response surface methodology, liver injury

## Abstract

The consumption of herbal teas has become popular in recent years due to their attractive flavors and outstanding antioxidant properties. The Five-Golden-Flowers tea is a herbal tea consisting of five famous edible flowers. The effects of microwave-assisted extraction parameters on the antioxidant activity of Five-Golden-Flowers tea were studied by single-factor experiments, and further investigated using response surface methodology. Under the optimal parameters (53.04 mL/g of solvent/material ratio, 65.52 °C, 30.89 min, and 500 W), the ferric-reducing antioxidant power, Trolox equivalent antioxidant capacity, and total phenolic content of the herbal tea were 862.90 ± 2.44 µmol Fe^2+^/g dry weight (DW), 474.37 ± 1.92 µmol Trolox/g DW, and 65.50 ± 1.26 mg gallic acid equivalent (GAE)/g DW, respectively. The in vivo antioxidant activity of the herbal tea was evaluated on alcohol-induced acute liver injury in mice. The herbal tea significantly decreased the levels of aspartate aminotransferase, total bilirubin, and malonaldehyde at different doses (200, 400, and 800 mg/kg); improved the levels of liver index, serum triacylglycerol, and catalase at dose of 800 mg/kg. These results indicated its role in alleviating hepatic oxidative injury. Besides, rutin, chlorogenic acid, epicatechin, gallic acid, and *p*-coumaric acid were identified and quantified by high performance liquid chromatography (HPLC), which could contribute to the antioxidant activity of the herbal tea.

## 1. Introduction

The oxidative damage induced by excessive free radicals is responsible for the pathogenesis of chronic diseases, which has attracted considerable public concern [1]. Natural products could be utilized in the prevention and treatment of chronic diseases, such as cancer and cardiovascular disease, partly due to their potent antioxidant activities [2,3].

Several herbal teas were found to possess abundant natural antioxidants which can strengthen the antioxidant defense system and have the potential to prevent diseases induced by oxidative stress [4,5]. Rose (*Rosa rugosa*), osmanthus (*Osmanthus fragrans*), chrysanthemum (*Flos chrysanthemi*), honeysuckle (*Lonicera japonica*), and jasmine (*Jasminum sambac*) are five important flowers with various health benefits and have been consumed as herbal teas for a long time [6]. The Five-Golden-Flowers tea is a herbal tea, and consists of these five flowers, with satisfied sensory properties and plentiful antioxidants. 

In this study, the effects of microwave-assisted extraction parameters on the antioxidant activity of Five-Golden-Flowers tea were studied. Furthermore, the alcohol-induced acute liver injury in Kunming mice model was utilized to evaluate the in vivo antioxidant and hepatoprotective activities of the herbal tea. Additionally, the phenolic compounds presented in the herbal tea were identified and quantified by high performance liquid chromatography (HPLC). This study will facilitate the exploration of the herbal tea as a good source of natural antioxidants for preventing several diseases induced by oxidative stress.

## 2. Results and Discussion

### 2.1. Results of Single-Factor Tests

Several mechanisms are involved in the antioxidant activities of antioxidants, such as free radical scavenging, metal ions chelating, and stimulating endogenous antioxidant compounds [7]. Thus, it is necessary to detect the in vitro antioxidant capacities through several methods with various testing principles [8]. The ferric-reducing antioxidant power (FRAP) and Trolox equivalent antioxidant capacity (TEAC) are two common assays to rapidly evaluate the antioxidant capacities [9]. The FRAP assay detects the ferric ions reducing ability of the herbal tea, while the TEAC assay detects the free radical scavenging activity. 

**Figure 1:**
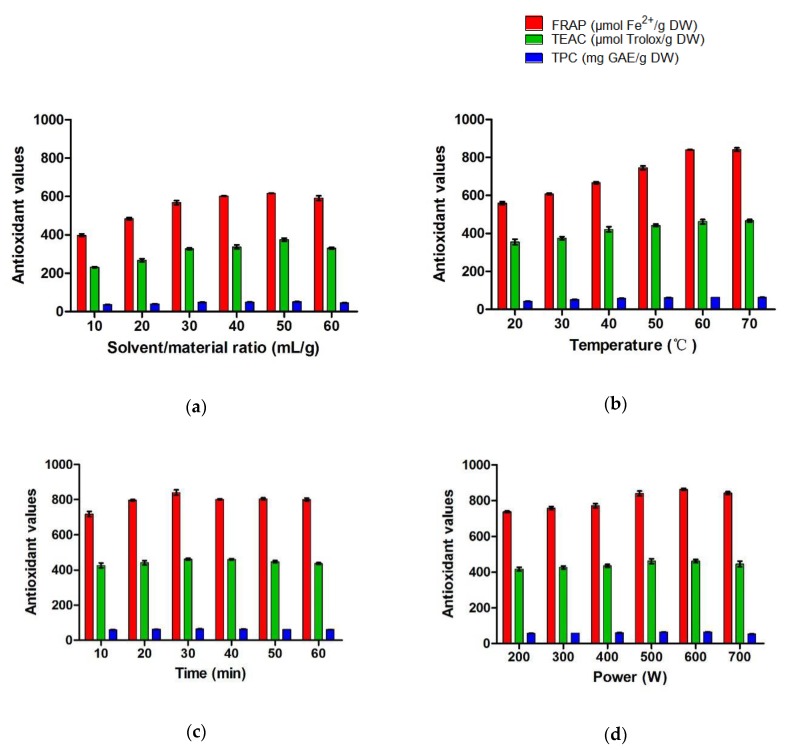
Effects of solvent/material ratio (**a**), temperature (**b**), time (**c**), and microwave power (**d**) on antioxidant values. GAE: gallic acid equivalent; DW: dry weight.

Extraction procedure can influence the yield, composition, and biological activity of the extract [10,11]. The influence of extraction parameters on antioxidant capacities measured by FRAP and TEAC, as well as total phenolic content (TPC) was explored in single-factor tests (Figure 1). 

The impacts of ratio of solvent to material (S/M ratio) on antioxidant values were studied when other parameters were fixed as 30 °C, 30 min, and 500 W (Figure 1a). The antioxidant values increased significantly from 10 to 40 mL/g of S/M ratio. From 40 to 50 mL/g of S/M ratio, the FRAP value increased non-significantly, while the values of TEAC and TPC increased significantly. Significant decreases of the antioxidant values were observed from 50 to 60 mL/g of S/M ratio. At the optimal 50 mL/g of S/M ratio, the mass transfer probably reached the maximum. 

As shown in Figure 1b, the antioxidant values varied greatly depending on temperature under conditions of 50 mL/g of S/M ratio, 30 min, and 500 W. The antioxidant values markedly improved as the temperature increased from 20 to 60 °C, and kept almost constant when the temperature continued to rise. To save energy, 60 °C was selected in subsequent tests. 

Under a certain procedure (50 mL/g of S/M ratio, 60 °C, and 500 W), the antioxidant values improved from 10 to 30 min extraction, and showed a downward trend with further increasing time (Figure 1c), which might cause the degradation of antioxidants [12]. Hence, 30 min is optimal for the extraction procedure.

The sample was extracted under 50 mL/g of S/M ratio, 60 °C, and 30 min, with different levels of microwave power (Figure 1d). Although the antioxidant values reached the peak at 600 W, there was no significant difference between 500 and 600 W. In consideration of energy-saving, 500 W was more suitable in the extraction of antioxidants. 

### 2.2. Results of Response Surface Methodology Tests

#### 2.2.1. Results of Central Composite Design

Taking into consideration the results of single-factor tests, three dominant variables, i.e., S/M ratio, temperature, and time were further optimized by response surface methodology (RSM) using central composite design (CCD). The ranges of these 3 process variables were designed as S/M ratio (X_1_; 40, 50, and 60 mL/g), temperature (X_2_; 50, 60, and 70 °C), and time (X_3_; 20, 30, and 40 min). The actual and coded levels of the 3 independent variables, and their corresponding response values (actual and predicted) are listed in Table 1. 

#### 2.2.2. Model Fitting

The actual values of FRAP, TEAC, and TPC under 20 experimental combinations varied depending on the variance of extraction procedure, and were fitted into Equations (1)–(3) to evaluate the relationship between variables (X_1_—S/M ratio, X_2_—temperature, X_3_—time) and each response with non-significant items being removed:

Y_FRAP_ = 840.61 + 16.72 X_1_ + 35.87 X_2_ − 25.88 X_1_^2^ − 31.38 X_2_^2^ − 37.42 X_3_^2^ (1)

Y_TEAC_ = 461.70 + 8.90 X_1_ + 29.91 X_2_ − 13.63 X_1_^2^ − 24.34 X_2_^2^ − 22.69 X_3_^2^ (2)

Y_TPC_ = 64.40 + 1.36 X_1_ + 3.07 X_2_ − 1.09 X_1_ X_2_ − 2.19 X_1_^2^ − 3.08 X_2_^2^ − 2.55 X_3_^2^ (3)

As shown in Table 2, the analysis of variance (ANOVA) of FRAP indicated that the model was significant (F = 23.92, *p* < 0.0001). The non-significance (*p* = 0.4336) of lack-of-fit testing further verified the suitability of the model. Besides, the determination coefficient value (*R*^2^) of 0.9556 suggested that 95.56% of the variation could be explained by the fitted model. Furthermore, the adjusted *R*^2^ of 0.9156 was closed to *R^2^*, which proved the high correlation between the actual responses and the predicted responses. In a similar way, the models of TEAC and TPC were also suitable to predict the real relationship between the independent variables and the response. 

#### 2.2.3. Graphical Analysis

**Figure 2:**
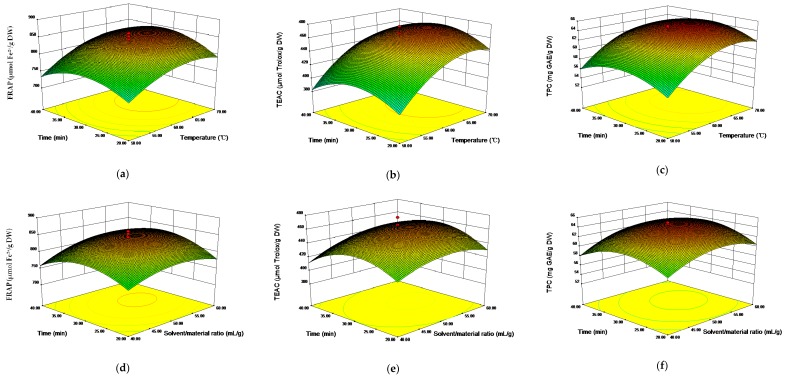

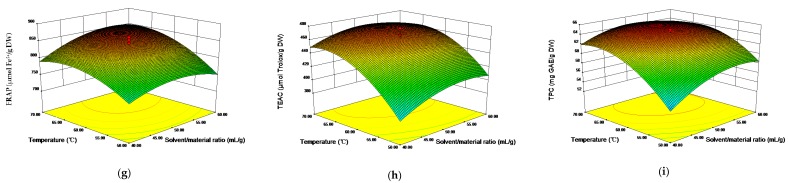
Graphical analysis of effects of temperature and time on FRAP (**a**), TEAC (**b**), and TPC (**c**); solvent/material ratio and time on FRAP (**d**), TEAC (**e**), and TPC (**f**); solvent/material ratio and temperature on FRAP (**g**), TEAC (**h**), and TPC (**i**).

The 3D response surfaces plots visually illustrated the relationships between independent variables and response values (Figure 2). The interactions of time and temperature on the FRAP, TEAC, and TPC values were plotted at a fixed extraction S/M ratio of 50 mL/g (Figure 2a–c). The increase of temperature obviously elevated the response values, and reached the peak at 65 °C, while the response values increased slightly with the increase of time from 20 min to 30 min. Figure 2d–f shows the interactions between time and S/M ratio on the response values at 60 °C. The effect of time on the response values was similar to that shown in Figure 2a–c. As the S/M ratio increased from 40 to 50 mL/g, the response values improved markedly, then decreased as the S/M ratio continued to increase. Figure 2g–i plots the interactions of S/M ratio and extraction temperature on the response values at 30 min, which followed a similar trend with those results in Figure 2a–f. Considering the results of response surfaces plots and the ANOVA in Table 2, it could be concluded that S/M ratio and extraction temperature significantly affected the response values.

#### 2.2.4. Optimal Extraction Parameters and Responses

Under optimal parameters (53.04 mL/g of S/M ratio, 65.52 °C, 30.89 min, and 500 W), the FRAP, TEAC, and TPC values were 862.90 ± 2.44 µmol Fe^2+^/g dry weight (DW) of the herbal tea, 474.37 ± 1.92 µmol Trolox/g DW, and 65.50 ± 1.26 mg gallic acid equivalent (GAE)/g DW, respectively, which were in accordance with the predicted ones (FRAP = 856.59 µmol Fe^2+^/g DW, TEAC = 471.44 µmol Trolox/g DW, TPC = 65.18 mg GAE/g DW).

### 2.3. Comparison of Different Extraction Methods

The extraction efficiency of microwave-assisted extraction (MAE) was compared with traditional decocting and Soxhlet extraction in terms of time, temperature, solvent, and extraction yields (Table 3). MAE allowed a greater yield of natural antioxidants in herbal tea in comparison with decocting method. The FRAP, TEAC, and TPC values of MAE extract were 1.19, 1.27, and 1.18 times higher than those of decocting method. The high efficiency of MAE could be attributed to ionic conduction and dipole rotation provided by microwave energy, which could rapidly increase the inner temperature of the plant cells [13,14,15]. Then, the sudden heat facilitated the fracture of cell walls and ultimately accelerated the release of antioxidants from the plant matrix into the solution [16,17,18]. 

Furthermore, MAE obtained similar antioxidant yields in comparison with Soxhlet extraction in relatively shorter time and lower temperature, which might protect some thermal unstable components from decomposition [19,20]. It should be pointed out that prolonged microwave irradiation time could also induce the degradation of some antioxidants as shown in Figure 1c. Thus, the optimization of the MAE parameters was essential for warranting the maximal extraction of natural antioxidants from herbal tea. Additionally, compared with Soxhlet extraction, MAE was conducted without organic solvent, which was green and economic. 

### 2.4. Correlations between FRAP, TEAC, and TPC

**Figure 3:**
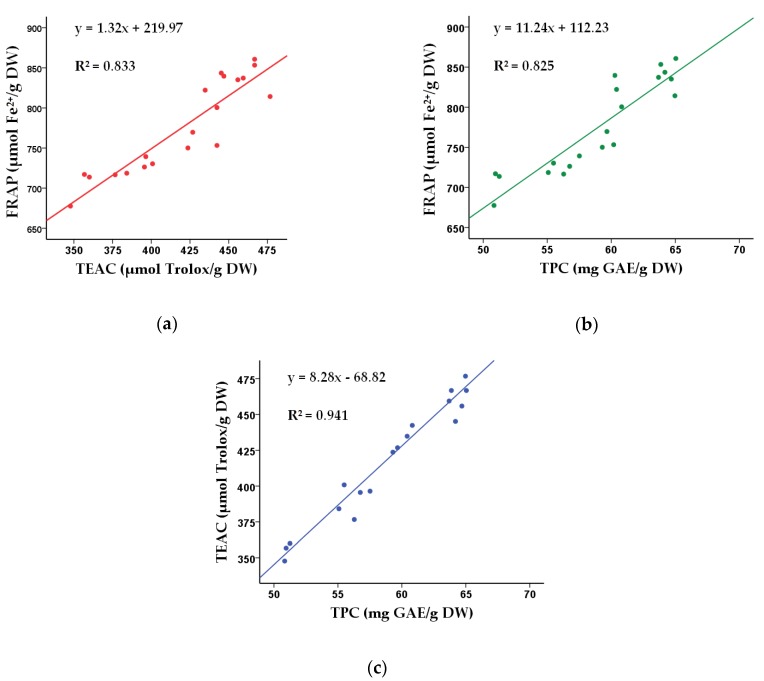
Correlations between values of FRAP and TEAC (**a**), FRAP and TPC (**b**), TEAC and TPC (**c**).

The correlations of the actual values of FRAP, TEAC, and TPC presented in Table 1 were analyzed (Figure 3). The strong linear correlation between FRAP and TEAC (*R*^2^ = 0.833) implied that antioxidants in the herbal tea possessed both reducing Fe^3+^ to Fe^2+^ and scavenging 2,2′-azino-bis(3-ethylbenothiazoline-6-sulphonic acid) (ABTS) free radicals activities. Besides, the strong linear correlation between TPC and FRAP/TEAC (FRAP vs. TPC, *R*^2^ = 0.825; TEAC vs. TPC, *R*^2^ = 0.941) indicated that phenolic compounds could be responsible for the reducing Fe^3+^ to Fe^2+^ and scavenging ABTS free radicals activities of the herbal tea. 

### 2.5. Results of Animals Study

#### 2.5.1. Hepatoprotective Effects of the Herbal Tea against Alcohol-Induced Injury

The liver index (ratio of liver weight and body weight), and the levels of serum aspartate transaminase (AST), alanine transaminase (ALT), alkaline phosphatase (ALP), total bilirubin (TBIL), as well as the contents of serum and liver triglyceride (TG) were measured to assess effects of the herbal tea on liver injury (Table 4). AST and ALT are aminopherases concentrating in hepatocyte cytoplasm which will leak into plasma as hepatocytes are damaged by alcohol and its metabolites [21]. The impairment of hepatic conjugated bilirubin excretion causes the increase of serum TBIL, which reflects the functional lesion of the liver [22]. As shown in Table 4, the liver index (*p* < 0.01) and serum levels of AST (*p* < 0.01), ALT (*p* < 0.05), TBIL (*p* < 0.05), and serum TG (*p* < 0.05) were increased significantly in the model group compared with the control group, which indicated the liver injury induced by alcohol. The results showed that all three doses of herbal tea (200, 400, and 800 mg/kg) ameliorated the levels of AST and TBIL significantly. Moreover, high-dose treatment markedly decreased liver index compared with the model group (*p* < 0.05). Consumption of alcohol results in deposition of hepatic TG, which will be delivered into blood and cause the increase of serum TG. So the concentrations of TG in serum and liver need to be measured [23]. The value of serum TG was lower at dose of 800 mg/kg (*p* < 0.05), indicating the role of the herbal tea in improving lipid metabolic abnormality induced by alcohol consumption [24]. Nevertheless, the difference in levels of ALP (target of hepatobiliary effect and cholestasis) and liver TG in 5 groups were non-significant. 

#### 2.5.2. The In Vivo Antioxidant Activity of the Herbal Tea

The antioxidant activity involves complicated mutual actions between the antioxidants and organisms [25], thus in vivo experiments need to be conducted. In this study, we employed an alcohol-induced acute liver injury model to analyze the in vivo antioxidant activities of the herbal tea. The decisive role of oxidative stress in alcohol-induced liver injury has been reported by substantial literature [26,27]. Excessive alcohol consumption is a major risk factor in the disorder of hepatic function, as it induces the generation of free radicals, consumes cellular antioxidants, and leads to hepatocellular oxidative stress [28]. 

The activity of antioxidant enzymes superoxide dismutase (SOD) and catalase (CAT), and the contents of glutathione (GSH) and malondialdehyde (MDA) in liver are important indices reflecting antioxidant activity in vivo [29,30]. It has been reported that alcohol induced the damage of antioxidant defense system presented as the reduction of the activity of main antioxidant enzymes such as SOD (remove superoxide) and CAT (remove H_2_O_2_) [29]. Besides, the content of MDA (product of lipid peroxidation induced by free radicals) is an indirect indicator of the degree of the liver peroxidation damage [30]. 

As shown in Table 5, the value of MDA was significantly increased (*p* < 0.01) and the levels of SOD and CAT were significantly decreased (*p* < 0.05) 6 h after consumption of alcohol when compared with the control group, indicating that alcohol impaired the antioxidant activity of liver. In comparison with the model group, all 3 doses of herbal tea (200, 400, and 800 mg/kg) significantly inhibited the increase of MDA value, and the high dose of the herbal tea significantly increased the CAT activity (*p* < 0.05). Furthermore, the content of GSH and the activity of SOD increased in comparison with the model group, though the differences were insignificant. These results indicated that the hepatoprotective actions of the herbal tea might be attributed to its antioxidant activity.

#### 2.5.3. Histopathological Evaluation 

**Figure 4:**
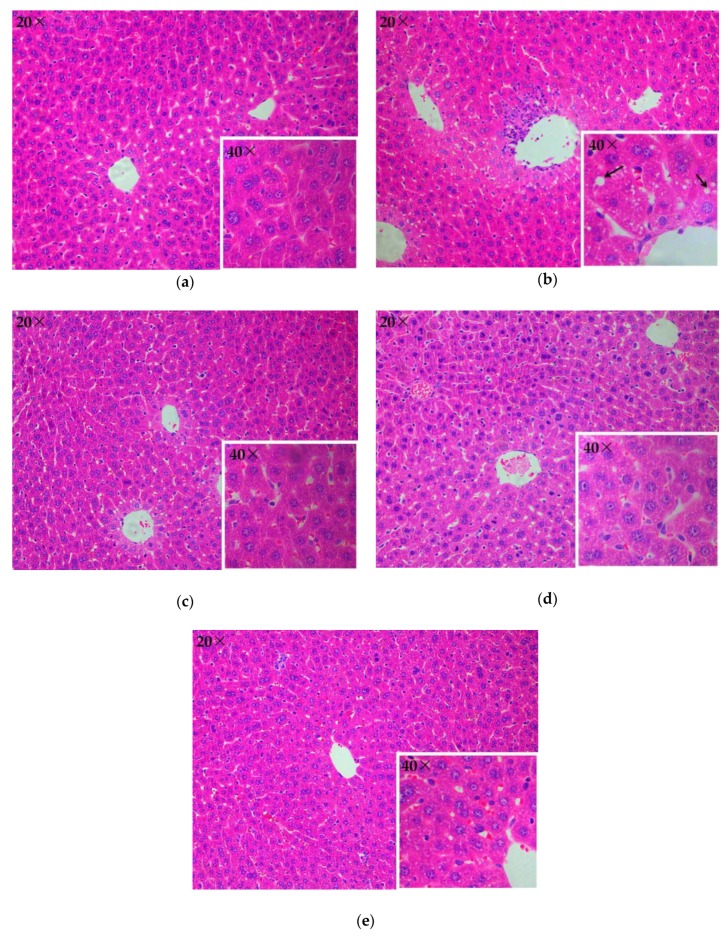
Hematoxylin-eosin stained liver sections of the control group (**a**), model group (**b**), 200 mg/kg group (**c**), 400 mg/kg group (**d**), and 800 mg/kg group (**e**). Arrow: lipid droplet.

Histopathological evaluation further revealed the protective role of the herbal tea against alcohol-induced liver injury (Figure 4). The control group showed no visible lesions (Figure 4a). In comparison with the control group, there were obvious pathologic changes (lipid droplets accumulation) in the model group. The liver sections of the model group were observed with 40× objective lens, and lipid droplets were found in less than 25% liver cells (Figure 4b). However, alcohol-induced lesion was attenuated in the 3 treatment groups (Figure 4c–e), displaying lighter steatosis as compared with the model group.

### 2.6. Analysis of Phenolic Compounds by HPLC

**Figure 5:**
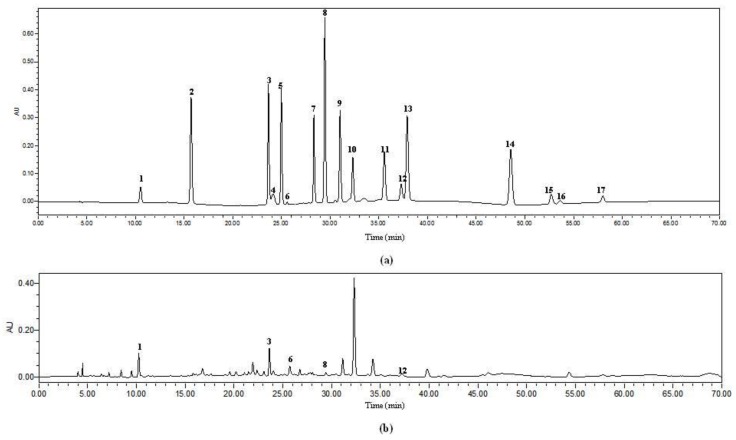
Chromatograms under 276 nm of standard phenolic compounds (**a**) and the herbal tea extract obtained using MAE under optimal parameters (**b**). 1. gallic acid; 2. protocatechuic acid; 3. chlorogenic acid; 4. cyanidin-3-glucoside; 5. caffeic acid; 6. epicatechin; 7. catechine; 8. *p*-coumaric acid; 9. ferulaic acid; 10. melatonin; 11. 2-hydroxycinnamic acid; 12. rutin; 13. resveratrol; 14. daidzein; 15. equol; 16. quercetin; 17. genistein.

Phenolic compounds in the extract of the herbal tea obtained using MAE under optimal parameters were identified and quantified by HPLC. Chromatograms under 276 nm of standard phenolic compounds and the herbal tea extract were presented in Figure 5. Five phenolic compounds, i.e., rutin, chlorogenic acid, epicatechin, gallic acid, and *p*-coumaric acid, were detected, with contents of 1.55 ± 0.20 mg/g DW, 1.44 ± 0.00 mg/g DW, 0.71 ± 0.06 mg/g DW, 0.67 ± 0.02 mg/g DW, and 0.10 ± 0.02 mg/g DW, respectively. 

Rutin, the most abundant phenolic compound detected in the herbal tea, has potent in vitro antioxidant activity measured by various assays [31], and could increase the antioxidant status in mouse liver [32]. Moreover, a study demonstrated that chlorogenic acid treatment attenuated alcohol-induced liver injury through suppressing oxidative stress [33]. Besides, the other 3 phenolic compounds have also shown potent antioxidant activities [34]. Given these, the 5 phenolic compounds might be the bioactive components responsible for the antioxidant and hepatoprotective activity.

## 3. Materials and Methods 

### 3.1. Sample Preparation

Dried flowers of rose, osmanthus, chrysanthemum, honeysuckle, and jasmine purchased from supermarket in Guangzhou, China were formulated in proportion of 28.57%, 28.57%, 21.43%, 14.29%, and 7.14%, respectively. The mixture was ground into particles using a grinder (RS-FS500B; Royalstar Co., Ltd., Hefei, Anhui, China), and then filtered through a 100 meshes sieve. 

### 3.2. Chemicals

TPTZ (2,4,6-tri(2-pyridyl)-*s*-triazine), Folin & Ciocalteu’s phenol, Trolox (6-hydroxy-2,5,7,8-tetramethylchromane-2-carboxylic acid), gallic acid, ABTS (2,2′-azino-bis(3-ethylbenothiazoline-6-sulphonic acid) diammonium salt), and phenolic standards (such as rutin and epicatechin) were products of Sigma-Aldrich (Saint Louis, MO, USA). Chromatography-grade formic acid and methanol were purchased from Kermel Chemical Factory (Tianjin, China). All the other regents (such as sodium carbonate anhydrous) were of analytical grade, and were produced by Damao Reagent Factory (Tianjin, China). The total protein (TP), TG, MDA, GSH, SOD, and CAT kits were purchased from Nanjing Jiancheng Bioengineering Institute (Nanjing, China). 

### 3.3. Microwave-Assisted Extraction

MAE was executed using a microwave extraction device (XH-100A; Xianghu Instrumental Company, Beijing, China). The sample (0.200 g) was extracted using a certain volume of distilled water in a tube. The tube was put into a water bath equipped with a temperature monitor. Then, the sample was extracted under pre-set process parameters, which was controlled by the software of the device. In the single-factor tests, the experimental parameters were set as: S/M ratio (10, 20, 30, 40, 50, and 60 mL/g), temperature (20, 30, 40, 50, 60, and 70 °C), time (10, 20, 30, 40, 50, and 60 min), and microwave power (200, 300, 400, 500, 600, and 700 W). Then, the S/M ratio, time, and temperature were further optimized using RSM, and their designed levels were displayed in Table 1, while the microwave power was fixed at 500 W. After extraction, the mixture was centrifugated for 30 min at 4200 g, and the supernatant was gathered.

### 3.4. Decocting Extraction

The 0.200 g powdered sample was immersed in 10.608 mL distilled water with stirring, and extracted for 30.89 min at 65.52 °C in a water bath shaker. After centrifugation (4200 g, 30 min), the supernatant was collected. 

### 3.5. Soxhlet Extraction

The Soxhlet extraction was executed according to the procedure previously reported [19]. The sample (1.000 g) was extracted by 200 mL of 50% (*v*/*v*) ethanol aqueous solution in a Soxhlet extractor at 95 °C water bath for 4 h. After extraction, the solution was collected.

### 3.6. Measurement of Antioxidant Capacities and Total Phenolic Contents 

The FRAP, TEAC, and TPC of the herbal tea were measured based on procedures previously published [4], which were stated as µmol Fe^2+^/g DW, µmol Trolox/g DW, and mg GAE/g DW, respectively.

### 3.7. Optimization of Extraction Parameters

The influences of 4 process variables on antioxidant values (FRAP, TEAC, and TPC) were evaluated in single-factor tests. Then, 3 selected dominant factors were further optimized in following response surface methodology (RSM) by Design Expert 8.0.6 (Stat-Ease Inc., Minneapolis, MN, USA). 

A total of 20 runs designed according to the three-variable and five-level CCD were consisted of 8 combinations of factorial points, 6 combinations of axial points, and 6 replicates of center point. The variation of response value versus the 3 dominant variables (X_1_, X_2_, and X_3_) was fitted into following response surface quadratic model:

Y = ∑β_i_X_i_ + ∑β_ii_X_i_^2^ + ∑β_ij_X_i_X_j_ + β_0_ (4)

where Y was the response value (FRAP, TEAC, and TPC value); X_i_ and X_j_ were the independent variables; β_i_, β_ii_, β_ij_, and β_0_ were the coefficients of the linear, quadratic, interactive, and constant terms, respectively. 

ANOVA was carried out to test the adequacy of the fitted model at a significant level of *p* < 0.05. The 3D surface plots were utilized to visualize the individual and interactive effects of independent variables on the response value. Besides, the verified experiment was conducted to verify the accuracy of the fitted model. 

### 3.8. Animal Study

The supernatant obtained under optimal MAE procedure was evaporated to dryness by vacuum rotary evaporator. Then, the dried crude extract was dissolved in distilled water at concentrations of 10, 20, and 40 g/L.

The 30 male Kunming mice weighing 18–22 g were purchased from Experimental Animal Center of Sun Yat-sen University, and housed in SPF animal room (12 h light/dark cycle, 22 ± 0.5 °C, 40–60% relative humidity). All procedures were strictly executed according to the principles of “laboratory animal care and use” approved by Sun Yat-sen University (No. 2017-011; 21 November 2017). Mice were randomly divided into 5 groups (6 mice each), namely control group, model group, and 3 treatment groups. 

The 3 treatment groups were fed intragastrically with 0.2 mL/10 g body weight of herbal tea at different doses (200, 400, and 800 mg/kg, according to the literature [23]) for 7 days, while the control and model groups were treated with corresponding distilled water. The model and 3 treatment groups were administrated with 52% (*v*/*v*) alcohol (10 mL/ kg body weight, i.g.) 30 min after the last administration, while the control group was ingested with corresponding distilled water. All animals were weighed and anesthetized 6 h later. 

The blood samples were collected after removing eyeball, then centrifuged at 3600 g for 10 min twice. The levels of AST, ALT, ALP, TBIL, and TG of the separated serums were measured by chemistry analyzer (AU5821; Beckman Coulter K.K., Tokyo, Japan). The liver was harvested and weighed. One piece of the liver was immersed in 4% (*w*/*v*) paraformaldehyde, embedded in paraffin, and sliced for staining with hematoxylin-eosin (H&E). The 0.200 g liver tissue was homogenized in 1.8 mL ice-cold 0.9% NaCl solution. The homogenate (10%, *w*/*v*) was centrifuged at 2500 *g* for 10 min to obtain supernatant, which was used for determining the levels of TP, TG, MDA, GSH, SOD, and CAT according to kits instructions. The significant increase of MDA content, and decreases of GSH, SOD, and CAT activities in the model group indicate the occurrence of alcohol-induced oxidative damage in liver [22].

### 3.9. HPLC Assay

The phenolic profile of the herbal tea, which was acquired using the optimal parameters, were identified and quantified according to the method reported by Cai et al. with little modifications [35]. HPLC system was equipped with photodiode array detector (PAD) (Waters, Milford, MA, USA) and Agilent Zorbax Extend-C18 column (5 µm, 4.6 mm × 250 mm). The HPLC procedure was set as follows: mobile phase A of 0.1% formic acid in water; mobile phase B of methanol; temperature of 40 °C; flow rate of 0.8 mL/min; elution gradient of 95% A (0 min), 80% A (15 min), 70% A (20 min), 63% A (25 min), 60% A (40 min), 50% A (60 min), 50% A (65 min), 95% A (65.1 min), 95% A (70 min). The retention time, UV-vis spectrum, and peak area (under the maximal absorption wavelength) were compared with those of standards to identify and quantify phenolic compounds in the herbal tea, and the content was expressed as mg/g DW.

### 3.10. Data Analysis

All tests were conducted in triplicate, and the values were expressed as mean ± SD (standard deviation). Data analysis was performed using SPSS 20.0. For comparison between more than two groups, one-way ANOVA was used, and followed by a LSD post hoc test, with a significant level of 0.05. For correlations between values of FRAP, TEAC, and TPC, Pearson test was utilized.

## 4. Conclusions

The optimized extraction parameters (53.04 mL/g of S/M ratio, 65.52 °C, 30.89 min, and 500 W) guaranteed the maximal yield of antioxidants from Five-Golden-Flowers tea. The in vitro antioxidant assays indicated that the herbal tea possessed considerable ABTS radical scavenging activities, Fe^3+^ reducing power, as well as high content of phenolic compounds. The in vivo animal experiment illustrated that the herbal tea exerted antioxidant properties against alcohol-induced oxidative damage in liver via enhancing antioxidant enzyme activity and reducing lipid peroxidation. Moreover, the phenolic compounds, the major antioxidant contributors of the herbal tea, were detected using HPLC, with rutin, chlorogenic acid, epicatechin, gallic acid, and *p*-coumaric acid being identified and quantified. All of the results supported the role of Five-Golden-Flowers tea as a potential source of natural antioxidants, which could be utilized as functional foods for the prevention of oxidative stress-induced diseases.

## Figures and Tables

**Table 1:** Central composite design, actual and coded levels of independent variables, and corresponding actual and predicted values of responses.

Run	S/M Ratio (X_1_, mL/g)	Temperature (X_2_, °C)	Time (X_3_, min)	FRAP (Y_1_, µmol Fe^2+^/g DW)		TEAC (Y_2_, µmol Trolox/g DW)		TPC (Y_3_, mg GAE/g DW)	
				Actual	Predicted	Actual	Predicted	Actual	Predicted
1	33.18 (−1.68)	60 (0)	30 (0)	730.34	739.28	400.82	408.20	55.49	55.93
2	40 (−1)	50 (−1)	40 (1)	717.07	695.95	356.78	351.29	50.95	50.97
3	60 (1)	50 (−1)	20 (−1)	718.60	707.21	384.12	382.34	55.08	55.29
4	60 (1)	70 (1)	20 (−1)	769.67	787.98	426.78	430.07	59.66	59.55
5	50 (0)	60 (0)	13.18 (−1.68)	726.33	723.71	395.54	398.51	56.75	56.96
6	50 (0)	60 (0)	30 (0)	837.24	840.61	459.39	461.70	63.69	64.40
7	50 (0)	60 (0)	30 (0)	843.60	840.61	445.18	461.70	64.19	64.40
8	50 (0)	60 (0)	30 (0)	860.73	840.61	466.76	461.70	65.04	64.40
9	50 (0)	76.82 (1.68)	30 (0)	822.17	812.19	434.81	443.16	60.41	60.85
10	50 (0)	60 (0)	46.82 (1.68)	739.25	745.84	396.43	396.57	57.51	57.43
11	60 (1)	50 (−1)	40 (1)	716.60	720.97	376.63	386.82	56.28	56.62
12	50 (0)	43.18 (−1.68)	30 (0)	677.58	691.54	347.78	342.54	50.84	50.52
13	50 (0)	60 (0)	30 (0)	814.25	840.61	476.77	461.70	64.97	64.40
14	66.82 (1.68)	60 (0)	30 (0)	800.50	795.53	442.40	438.13	60.81	60.50
15	60 (1)	70 (1)	40 (1)	839.67	834.47	446.86	440.56	60.28	60.29
16	40 (−1)	70 (1)	20 (−1)	753.28	746.10	442.40	430.01	60.19	59.77
17	40 (−1)	50 (−1)	20 (−1)	713.73	716.12	359.99	364.09	51.25	51.15
18	50 (0)	60 (0)	30 (0)	853.40	840.61	466.76	461.70	63.87	64.40
19	50 (0)	60 (0)	30 (0)	835.10	840.61	455.90	461.70	64.69	64.40
20	40 (−1)	70 (1)	40 (1)	750.08	758.66	423.65	423.23	59.30	59.00

FRAP: ferric-reducing antioxidant power; TEAC: Trolox equivalent antioxidant capacity; TPC: total phenolic content.

**Table 2:** ANOVA of the models of FRAP, TEAC, and TPC.

Source	FRAP					TEAC					TPC				
	Sum of Squares	df	Mean Square	F value	*p* Value	Sum of Squares	df	Mean Square	F Value	*p* Value	Sum of Squares	df	Mean Square	F Value	*p* Value
Model	61,370.96	9	6819.00	23.92	<0.0001	29,448.79	9	3272.09	29.03	<0.0001	417.00	9	46.33	169.39	<0.0001
X_1_	3818.96	1	3818.96	13.39	0.0044	1080.92	1	1080.92	9.59	0.0113	25.22	1	25.22	92.19	<0.0001
X_2_	17,571.62	1	17571.62	61.63	<0.0001	12,221.12	1	12,221.12	108.43	<0.0001	128.95	1	128.95	471.43	<0.0001
X_3_	591.38	1	591.38	2.07	0.1804	4.54	1	4.54	0.04	0.8450	0.27	1	0.27	0.97	0.3468
X_1_X_2_	1289.81	1	1289.81	4.52	0.0593	165.53	1	165.53	1.47	0.2534	9.48	1	9.48	34.67	0.0002
X_1_X_3_	575.62	1	575.62	2.02	0.1858	149.21	1	149.21	1.32	0.2767	1.13	1	1.13	4.14	0.0692
X_2_X_3_	535.63	1	535.63	1.88	0.2005	18.09	1	18.09	0.16	0.6971	0.17	1	0.17	0.63	0.4473
X_1_^2^	9652.51	1	9652.51	33.85	0.0002	2675.42	1	2675.42	23.74	0.0006	69.13	1	69.13	252.72	<0.0001
X_2_^2^	14,187.47	1	14,187.47	49.76	<0.0001	8540.02	1	8540.02	75.77	<0.0001	136.96	1	136.96	500.73	<0.0001
X_3_^2^	20,175.95	1	20,175.95	70.76	<0.0001	7416.22	1	7416.22	65.80	<0.0001	93.77	1	93.77	342.80	<0.0001
Residual	2851.23	10	285.12			1127.15	10	112.71			2.74	10	0.27		
Lack of fit	1537.40	5	307.48	1.17	0.4336	537.00	5	107.40	0.91	0.5400	1.09	5	0.22	0.66	0.6699
Pure error	1313.83	5	262.77			590.15	5	118.03			1.65	5	0.33		
Cor total	64,222.19	19				30,575.93	19				419.73	19			
*R* ^2^	0.9556					0.9631					0.9935				
Adjusted *R*^2^	0.9156					0.9300					0.9876				

**Table 3:** Comparison of three extraction methods.

Extraction Methods	Solvent	Temperature (°C)	Time	FRAP (μmol Fe^2+^/g DW)	TEAC (μmol Trolox/g DW)	TPC (mg GAE/g DW)
Decocting method	Distilled water	65.52	30.89 min	726.16 ± 1.25	372.61 ± 1.33	55.65 ± 0.39
Soxhlet extraction	50% ethanol	95	4 h	847.17 ± 2.36	479.15 ± 1.26	68.55 ± 0.32
MAE	Distilled water	65.52	30.89 min	862.90 ± 2.44	474.37 ± 1.92	65.50 ± 1.26

**Table 4:** Effects of the herbal tea on liver function.

Group	Liver Index (%)	AST (U/L)	ALT (U/L)	ALP (U/L)	TBIL (μmol/L)	Serum TG (mmol/L)	Liver TG (mmol/gprot)
Control	4.11 ± 0.13	115.25 ± 9.99	30.10 ± 6.50	318.67 ± 39.39	1.96 ± 0.40	0.88 ± 0.23	0.25 ± 0.05
Model	4.58 ± 0.32 ^##^	153.72 ± 22.18 ^##^	40.68 ± 7.82 ^#^	303.33 ± 49.79	2.38 ± 0.32 ^#^	1.28 ± 0.33 ^#^	0.27 ± 0.04
200 mg/kg	4.43 ± 0.15	128.62 ± 16.73 *	32.77 ± 5.63	305.67 ± 68.56	1.70 ± 0.39 **	0.99 ± 0.38	0.31 ± 0.04
400 mg/kg	4.51 ± 0.21	125.50 ± 17.81 *	38.68 ± 6.58	327.83 ± 69.15	1.98 ± 0.12 *	1.01 ± 0.32	0.31 ± 0.05
800 mg/kg	4.25 ± 0.22 *	126.92 ± 12.61 *	35.38 ± 6.10	338.33 ± 54.40	1.83 ± 0.23 **	0.91 ± 0.05 *	0.30 ± 0.04

^#^*p* < 0.05, ^##^ *p* < 0.01, the model group vs. the control group. * *p* < 0.05, ** *p* < 0.01, the treatment group vs. the model group. gprot: gram protein.

**Table 5:** Effects of herbal tea on MDA, GSH, SOD, and CAT.

Group	MDA (nmol/mgprot)	GSH (µmol/gprot)	SOD (U/mgprot)	CAT (U/mgprot)
Control	1.08 ± 0.42	8.39 ± 0.91	271.03 ± 15.79	62.46 ± 2.74
Model	1.96 ± 0.70 ^##^	5.88 ± 1.01	255.54 ± 13.71 ^#^	56.04 ± 8.33 ^#^
200 mg/kg	1.00 ± 0.25 **	5.99 ±1.09	264.78 ± 4.90	51.19 ± 2.25
400 mg/kg	0.87 ± 0.12 **	8.63 ± 0.64	256.33 ± 8.78	57.49 ± 4.58
800 mg/kg	0.82 ± 0.17 **	10.05 ± 0.69	262.06 ± 3.94	62.57 ± 4.18 *

^#^*p* < 0.05, ^##^ *p* < 0.01, the model group vs. the control group. * *p* < 0.05, ** *p* < 0.01, the treatment group vs. the model group.
